# The effects of antipsychotic discontinuation or maintenance on the process of recovery in remitted first-episode psychosis patients – A systematic review and meta-analysis of randomized controlled trials

**DOI:** 10.1192/j.eurpsy.2024.5

**Published:** 2024-01-22

**Authors:** Laurent Béchard, Charles Desmeules, Lauryann Bachand, Maxime Huot-Lavoie, Olivier Corbeil, Elizabeth Anderson, Sébastien Brodeur, Annie LeBlanc, Marie-France Demers, Sophie Lauzier, Marc-André Roy

**Affiliations:** 1 Faculty of Pharmacy, University Laval, Quebec, Canada; 2 University Institute of Mental Health of Quebec, CIUSSS-CN, Quebec, Canada; 3 CERVO Research Center, Quebec, Canada; 4 Faculty of Medicine, Laval University, Quebec, Canada; 5 King’s College London, London, UK; 6 VITAM - Centre de recherche en santé durable, Quebec, Canada; 7 CHU de Québec-Université Laval Research Centre, Quebec, Canada

**Keywords:** antipsychotic, drug tapering, first-episode psychosis, mental health recovery, meta-analysis, systematic review

## Abstract

**Background:**

The optimal duration of antipsychotic treatment following remission of first-episode psychosis (FEP) is uncertain, considering potential adverse effects and individual variability in relapse rates. This study aimed to investigate the effect of antipsychotic discontinuation compared to continuation on recovery in remitted FEP patients.

**Methods:**

CENTRAL, MEDLINE (Ovid), Embase, and PsycINFO databases were searched on November 2, 2023, with no language restrictions. RCTs evaluating antipsychotic discontinuation in remitted FEP patients were selected. The primary outcome was personal recovery, and secondary outcomes included functional recovery, global functioning, hospital admission, symptom severity, quality of life, side effects, and employment. Risk of bias was assessed using the Cochrane risk-of-bias tool 2, and the certainty of evidence was evaluated with GRADE. Meta-analysis used a random-effect model with an inverse-variance approach.

**Results:**

Among 2185 screened studies, 8 RCTs (560 participants) were included. No RCTs reported personal recovery as an outcome. Two studies measured functional recovery, and discontinuation group patients were more likely to achieve functional recovery (RR 2.19; 95% CIs: 1.13, 4.22; I^2^ = 0%; n = 128), although evidence certainty was very low. No significant differences were found in hospital admission, symptom severity, quality of life, global functioning, or employment between the discontinuation and continuation groups.

**Conclusions:**

Personal recovery was not reported in any antipsychotic discontinuation trial in remitted FEP. The observed positive effect of discontinuation on functional recovery came from an early terminated trial and an RCT followed by an uncontrolled period. These findings should be interpreted cautiously due to very low certainty of evidence.

## Introduction

Antipsychotics have proven efficacy in treating first-episode psychosis (FEP) as they allow achieving a rate of symptomatic remission at 12 months of approximately 54% [1]. However, uncertainty persists regarding the optimal duration of treatment beyond the initial episode, guidelines recommending treatment maintenance for periods of at least 1–5 years, depending on the guidelines considered [2, 3]. Two meta-analyses of randomized controlled trials (RCTs) published in 2018 found a higher rate of psychotic relapse in patients discontinuing treatment [4, 5]. However, the level of certainty for this finding was not presented in those articles. One narrative review in 2022, using the GRADE approach, concluded with low certainty of evidence that antipsychotic discontinuation increases the risk of psychotic relapse (risk difference 26%), although nearly half of the patients in whom treatment was discontinued did not experience relapse [3].

While relapse is a crucial outcome, these meta-analyses and narrative review provide no or few results for other patient important outcomes such as personal recovery, functional recovery, hospital readmissions, side effects associated with antipsychotic medications, global functioning, and overall quality of life (QoL), that also need to be considered while deciding to stop or maintain an antipsychotic. Personal recovery is generally defined as an individual’s journey toward achieving a fulfilling life despite the constraints imposed by mental illness, while functional recovery has been defined as a composite measure of achieving good functioning across domains of functioning as well as remission of psychotic symptoms [6–8].

The assessment of the certitude surrounding these alternative outcomes has remained conspicuously absent from prior reviews. Furthermore, it is worth noting that an additional study exploring antipsychotic discontinuation was published subsequent to the most recent review [9]. Despite its premature termination due to challenges in recruitment, this study remains a valuable source of potentially relevant data, particularly for outcomes where existing data are scarce, such as those beyond psychotic relapse [9].

Hence, the goal of the current systematic review was to summarize the results of published RCTs comparing the impact on recovery outcomes (main objective) and hospital readmissions, side effects associated with antipsychotic medications, global functioning, overall QoL, symptom severity and employment (secondary objective) between treatment discontinuation versus continuation following remitted FEP.

## Methods

We conducted a systematic review according to the Cochrane methodology and report its results following the Preferred Reporting Items for Systematic Reviews and Meta-Analysis (PRISMA) (Supplementary Appendix) [10]. The protocol for this review was not previously registered. Nevertheless, the review methods were designed *a priori* and underwent rigorous evaluation by two external specialists in meta-analysis from Laval University (Supplementary Appendix).

### Eligibility criteria

#### Types of participants

Participants, regardless of age, sex, gender, treatment setting, or nationality, and diagnosed with non-affective or affective FEP were considered. Patients had to be taking an antipsychotic medication and be in remission of their psychotic illness according to the Remission in Schizophrenia Working Group’s criteria or a clear definition must be stated [11]. Only studies with a minimum of 50% of patients with FEP were included.

#### Types of interventions

Studies using either discontinuation of a previously administered antipsychotic medication irrespective of the withdrawal method used (abrupt or gradual) were included. The antipsychotic may have been discontinued using placebo or simply stopping any medication. No restriction on the use of co-intervention was applied.

#### Comparator

Standard care was defined as maintenance of the antipsychotic treatment.

#### Outcomes

Studies reporting at least one measure of personal recovery, functional recovery, global functioning, QoL, employment, hospital readmission, psychosis symptoms, or adverse drug reactions (ADRs) were considered.

#### Type of studies

All RCTs, including either blinded or open-label studies, were considered. Only published and peer-reviewed articles were eligible. No time frame, publication year, or language criteria were applied.

### Search strategy and information sources

The electronic databases Cochrane Central Register of Controlled Trials (CENTRAL), MEDLINE (Ovid), Embase, and PsycINFO (Ovid) were searched without language or publication date restrictions. The research strategy was built by in information specialist using controlled vocabulary and text words related to the population, the intervention and the study design (Supplementary Appendix). Validated RCT filters were used for Embase, PsycINFO and MEDLINE [12–14]. Reference list of selected articles and identified review articles were searched manually. Study authors were contacted in cases of missing information, and the planned dates of coverage were from inception to November 2, 2023.

### Study records

Titles and abstracts were screened independently by pairs of reviewers for study eligibility using Covidence (www.covidence.org/); in case of disagreement, a third review author, screened the article, and then the three authors involved reached a consensus through further discussion. Full-text article assessment was made for all seemingly eligible titles and abstracts. All study exclusion decisions were documented.

### Data collection process

Pairs of reviewers independently extracted data from the selected studies onto a pre-piloted Excel spreadsheet; disagreements were discussed and resolved with a third review author. Missing information, such as means or standard deviations (SD), were handled by contacting the study authors.

### Data items

General information (authors, title, publication year, publication type, journal, and country); study characteristics (aims, design, start/end date, study duration, funding, and conflict of interest); participant characteristics (sample size, age, sex, setting, inclusion/exclusion criteria, ethnicity, diagnosis, comorbidities, duration of untreated psychosis, and remission duration); risk of bias assessed according to the Cochrane risk-of-bias tool for RCTs version 2 (ROB2) [15]; intervention/comparison characteristics (number of patients randomized in each group, antipsychotic agent and dose used prior to randomization in each group, discontinuation method, duration of intervention, co-interventions, and adverse events); and outcome measures (all primary and secondary outcome measures) were extracted from the included studies. For continuous outcomes, the scale used, means, SD, and result time point were collected. For dichotomous outcomes, the scale used (if any), the number of participants with the event in each group and the result time point were collected.

### Outcomes

Each outcome was assessed at the endpoint of the study. If assessments at multiple time points were available (i.e., assessment after an uncontrolled follow-up phase), the farthest was used to examine long-term effects.

#### Primary outcomes

Two recovery conceptualizations were analyzed: (1) personal recovery, a continuous outcome gauged by instruments like the questionnaire about the process of recovery, reflects an individual’s progress toward a fulfilling life amid mental illness [7, 8, 16], and (2) functional recovery, a dichotomous outcome measured by functional indices, represents overall functioning and symptom remission [6].

#### Secondary outcomes

The following secondary outcomes were considered: 1. Global functioning, that is, a measure summarizing functional across various domains into a single continuous variable, such as the Social and Occupational Functioning Assessment Scale (SOFAS) [17]. 2. QoL, which has been defined by the World Health Organization (WHO) as “an individual’s perception of their position in life in the context of the culture and value systems in which they live and in relation to their goals, expectations, standards, and concerns.” It has been operationalized as a continuous variable in instruments such as the WHO QoL scale (WHOQOL) [18]. 3. Hospital admission: Dichotomous outcome defined as the number of participants who were admitted to the hospital for psychiatric reasons. 4. Symptom severity: Continuous outcome assessed with a validated measure such as the Positive and Negative Syndrome Scale [19]. 5. Employment: Dichotomous outcome defined as the number of participants employed. 6. ADRs: Dichotomous outcome defined as the occurrence of any among a group of ADR.

### Risk of bias in individual studies

The risk of bias was assessed by pairs of reviewers independently during the data extraction using the RoB2 tool and the criteria described in the Cochrane Handbook [20, 21]. Disagreements were handled via discussion and, if needed, with the help of a third review author. Bias was assessed on a study level for each primary and secondary outcomes examined.

### Data synthesis

An initial analytical review assessed the feasibility of a meta-analysis by examining the study characteristics and outcomes. Quantitative synthesis required at least two studies reporting on a specific outcome; otherwise, the data were synthesized narratively. A random-effect model with an inverse-variance approach was made using Review Manager 5 to conduct the meta-analysis [21]. Continuous outcomes were evaluated using the standardized mean difference (SMD) with a 95% confidence interval (CI), as multiple different scales are often used in psychiatry. If studies reported a median as central tendency measure and a range as deviation measure, they were used to estimate the mean and SD [22]. If standard errors or CIs were reported as deviation measures, they were used to calculate an SD using the rules from the Cochrane Handbook [21]. Only data from unmodified scales with psychometric properties published in a peer-reviewed journal were considered in the meta-analysis to avoid bias [23]. Dichotomous outcomes were evaluated using relative risk (RR) with a 95% CI [24, 25]. Statistical heterogeneity was assessed using the I^2^ statistic [21]. Statistical heterogeneity was explored using subgroup analysis. Subgroup analyses were done to explore the effect of risk of bias (high, some concerns, and low risk), types of antipsychotics (first-generation, second-generation, or mixed), use of injectable antipsychotics (oral, long-acting injectable, mixed), mean antipsychotics defined daily doses (DDD) used (low [<0.9 DDD], normal [≥0.9 and <1.1 DDD] and high [≥1.1 DDD]), sex (female, male, or mixed), discontinuation methods (abrupt and gradual discontinuation), and remission duration (short [≤12 months], medium [>12 months and ≤18 months], and long duration [>18 months]) [3, 26, 27]. The selection of these subgroup analyses was informed by the understanding that the characteristics of antipsychotics, study quality, duration of remission, and discontinuation strategies may influence recovery outcomes.

### Confidence in cumulative evidence

The strength in the body of evidence was assessed by pairs of two reviewers independently using the GRADE approach for the primary and secondary outcomes [28]. Disagreements were handled via discussion and, if needed, with the help of a third review author.

## Results

**Figure 1.**
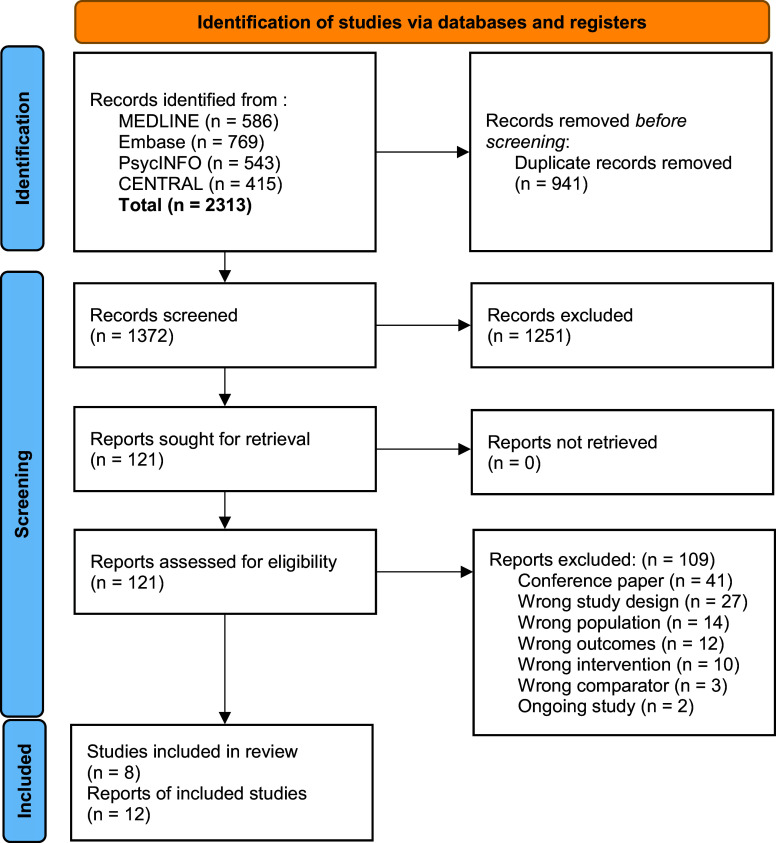
PRISMA flow diagram^a^. ^a^No records were obtained via manual searching of the reference lists of selected articles and identified review articles.

After the removal of duplicates, 1299 records were screened for eligibility (Figure 1). Five RCTs were excluded: one because of the lack of a maintenance of antipsychotic group [29], one because no outcome other than relapse was reported [30], one because it was not peer-reviewed [31], and two RCTs because they included fewer than 50% FEP patients [32, 33]. Eight studies were included (n = 560 participants), published from 1982 to 2022, with a median number of 37 participants per study (Table 1) [6, 9, 34–41]. Three studies were double blind placebo-controlled trials [34, 38, 39]. Five studies had an open-label design, two with a blinded assessment of outcomes [9, 40] and three without [36, 37, 41]. Follow-up durations were 1-year (five studies) [9, 34, 38, 39], 18 months follow-up (one study) [36], and 2 years (two studies) [37, 40, 41]. Two of these studies were followed by an uncontrolled follow-up phase, one for 5.5 years without blinded assessment [6, 36], and the other for 9 years with blinded assessment of outcomes [34, 35]. Only one study included psychotic mood disorders [38]. Two studies used first-generation antipsychotics exclusively, four studies used first- and second-generation antipsychotics, and two studies used second-generation antipsychotics exclusively. Remission duration before trial entry varied from 1 month to over 1-year. Two studies used abrupt discontinuation, while the others tapered off the antipsychotic over several weeks/months. Two studies assessed the impact of intermittent therapy, which permitted an escalation in antipsychotic dosage upon the reappearance of symptoms [40, 41].

**Table 1.** Characteristics of included studies

Study name (year); country, funding	Study design; (Trial duration)	Number of participants	Mean age/male/mean DDD/AP used	Remission duration	Intervention group and duration of taper	Maintenance group
Kane et *al.* (1982); USA, non-industry	Double blind placebo RCT (1 year)	Total: 28; Intervention: 17 Maintenance: 11	21.9 years/50%/NR/0% SGA	At least 1 month of remission	Placebo, 0 days	Fluphenazine IM 12.5–50 mg biweekly or oral 5–20 mg daily
McCreadie et *al.* (1989); UK, industry	Double blind placebo RCT (1 year)	Total: 15; Intervention: 7 Maintenance: 8	NR/NR/NR/0% SGA	No relapse for at least 12 months	Placebo, 0 days	Fluphenazine IM or pimozide once weekly
Gaebel et *al.* (2002); Germany, non-industry	Open label RCT with blinded assessment of outcomes (2 years)	Total: 115; Intervention: Prodrome based: 39 Crisis based: 40 Maintenance: 36	31 years/48%/0.7 DDD/12.2% SGA	Stable condition for at least 3 months	PI: AP dose reduction of 50% biweekly after clinical stabilization. AP reintroduced if prodromal symptoms. CI: AP dose reduction of 50% biweekly after clinical stabilization. AP only reintroduced if crisis occurs.	FGA oral or injectable or clozapine dose ≥100 mgCPZE
Gaebel et *al.* (2011); Germany, non-industry	Open label RCT (2 years)	Total: 44; Intervention: 21 Maintenance: 23	33.1 years/56.8%/0.5 DDD/65.9% SGA	No relapse for at least 12 months	AP tapering over 3 months	Risperidone ≤6 mg daily or haloperidol ≤6 mg daily
Boonstra et *al.* (2011); Netherlands, industry	Open label RCT (2 years)	Total: 20; Intervention: 11 Maintenance: 9	29.3 years/85%/0.4 DDD/95% SGA	Clinically stable for at least 12 months	AP tapering over 6–12 weeks	TAU (olanzapine, risperidone, quetiapine)
Wunderink et *al.* (2013); Netherlands, industry	Open label RCT (1.5 and 7 years)	Total: 131; Intervention: 65 Maintenance: 63	26.4 years/69.5%/0.3 DDD/95% SGA	Sustained remission for at least 6 months	AP tapering guided by symptom severity. AP reinitiation/dose increase allowed in the case of relapse.	TAU
Hui et *al.* (2018); Hong-Kong, industry	Double blind placebo RCT (1 and 10 years)	Total: 178; Intervention: 89 Maintenance: 89	24.2 years/45%/1.0 DDD/100% SGA	Relapse free in the last 12 months	Placebo cross-tapering 4–6 weeks	Quetiapine 400 mg daily cross-tapering 4–6 weeks
Stürup et *al.* (2022); Denmark, non-industry	Open label RCT with blinded assessment of outcomes (1 year)	Total: 29; Intervention: 14 Maintenance: 15	25.1 years/41.4%/1.0 DDD/100% SGA	Minimum of 3 months remission of psychotic symptoms	AP dose reduction 25% monthly	TAU

Abbreviations: AP, antipsychotic; CI, crisis-based intervention; FGA, First-generation antipsychotics; IM, intramuscular; mgCPZE, chlorpromazine equivalent dose; NR, not reported; PI, prodrome-based intervention; RCT, randomized controlled trial; SCZ, schizophrenia; SGA, second-generation antipsychotic; TAU, treatment as usual.

### Personal recovery

No identified RCT included personal recovery as an outcome.

### Functional recovery

**Figure 2.**
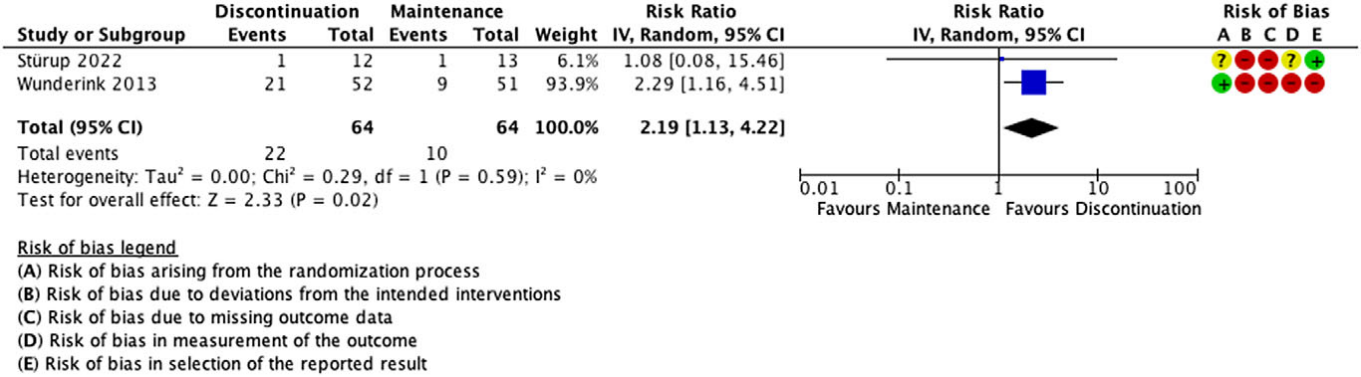
Effect of antipsychotic discontinuation in remitted first-episode psychosis patients on functional recovery*. Abbreviations: CI, confidence intervals; IV, inverse variance. *Functional recovery was defined as good functioning on all domains of the Groningen Social Disabilities Schedule (GSDS) and Andreasen’s criteria of symptomatic remission for 6 months.

Two RCTs (n = 128 participants) reported functional recovery defined as a composite outcome of good functioning on all domains of the Groningen Social Disabilities Schedule (GSDS) [42] and meeting Andreasen et al.’s criteria of symptomatic remission (for 6 months in the first trial and 3 in the second) (Figure 2) [6, 9, 11]. Patients assigned to the antipsychotic discontinuation group were more likely to achieve functional recovery (RR 2.19; 95% CIs: 1.13, 4.22; I^2^ = 0%) 1 and 7 years after discontinuation. Risk of bias was high in both studies because of the open nature of these trials, high attrition rates in one study, and high crossover rates in the other (i.e., continuation patients stopping treatment). Subgroup analyses could not be performed as only two studies were identified for this outcome. Certainty of the evidence for an increased rate of functional recovery was rated very low (Table 2).

**Table 2.** Summary of findings of outcomes following antipsychotic discontinuation

Outcomes	Illustrative comparative risks (95% CI)				
	Risk with maintenance	Risk with discontinuation	Relative effect [95% CI]	Number of participants (studies)	Certainty of the evidence (GRADE)
**Personal recovery – Not reported**	—	—	—	—	—
**Functional recovery** Follow-up: 1–7 years	16 per 100	**38 per 100** (20–73)	RR 2.19 [1.13, 4.22]	128 (2 RCTs)	⊕⊝⊝⊝^a^ ^,^^b^ ^,^^c^ ^,^^d^ ^,^^e^ **VERY LOW** – Risk of bias, indirectness, and imprecision
**Hospital admission** Follow-up: 1–2 years	8 per 100	**22 per 100** (11–46)	RR 2.01 [0.96, 4.22]	336 (6 RCTs)	⊕ ⊕ ⊝⊝^a,4,e^ **LOW** – Risk of bias and imprecision
**Positive symptoms** Follow-up: 1–10 years (various scales; higher score = worse)	—	SMD **0.28 higher** (0.21 lower to 0.77 higher)	—	350 (4 RCTs)	⊕⊝⊝⊝^a^ ^,^^b^ ^,^^c^ ^,^^d^ ^,^^e^ **VERY LOW** – Risk of bias, inconsistencies, indirectness, and imprecision
**Negative symptoms** Follow-up: 1–10 years (various scales; lower score = better)	—	SMD **0.03 lower** (0.36 lower to 0.30 higher)	—	350 (4 RCTs)	⊕⊝⊝⊝^a^ ^,^^b^ ^,^^c^ ^,^^d^ ^,^^e^ **VERY LOW** – Risk of bias, inconsistencies, indirectness, and imprecision
**Functioning** Follow-up: 1–10 years (various scales; lower score = worse)	—	SMD **0.32 lower** (0.91 lower to 0.27 higher)	—	314 (4 RCTs)	⊕⊝⊝⊝^a^ ^,^^b^ ^,^^c^ ^,^^d^ ^,^^e^ **VERY LOW** – Risk of bias, inconsistencies, indirectness, and imprecision
**Quality of life** Follow-up: 1–10 years (various scales; lower score = worse)	—	SMD **0.09 lower** (0.32 lower to 0.14 higher)	—	299 (4 RCTs)	⊕⊝⊝⊝^a^ ^,^^b^ ^,^^c^ ^,^^d^ ^,^^e^ **VERY LOW** – Risk of bias, indirectness, and imprecision
**Employment** Follow-up: 6.5–10 years	56 per 100	**64 per 100** (44–91)	RR 1.18 [0.82, 1.68]	242 (2 RCTs)	⊕⊝⊝⊝^a^ ^,^^b^ ^,^^c^ ^,^^d^ ^,^^e^ **VERY LOW** – Risk of bias, inconsistencies, indirectness, and imprecision

a Risk of bias: rated “serious” – Attrition was high and dropouts likely linked to symptom severity, functioning, and quality of life. Deviations from protocol due to open label design were likely and open-label studies most likely affected evaluation of outcomes. In subgroup analysis, studies with lower risk of bias showed different effect sizes than studies with high risk of bias.

b Inconsistency: rated “serious” – Subgroup analysis did not explain the heterogeneity and the I-square was higher than 50%.

c Indirectness: rated “serious” – The outcome was assessed after an uncontrolled period of multiple years in some studies. This diminished the certitude that the intervention is responsible of the observed effect.

d Imprecision: rated “serious” – Only few studies provided data for these outcomes, the confidence interval were large, sometimes ranging from harm to benefit.

e Publication bias: rated “no” – Publication bias was not detected.

### Global functioning

Four RCTs (n = 314 participants) reporting on global functioning were identified (Table 2) [6, 9, 35, 41]. Two RCTs used the Global Assessment of Functioning (GAF) scale [9, 41], two the GSDS [6, 9], one the SOFAS [35], one the Personal Social Performance scale [9], and one RCT the Role Functioning Scale [35]. As two studies used more than one scale to assess functioning, a post hoc decision was made to prioritize the use of measures closest to the global functioning construct (e.g., GAF, SOFAS scales). Antipsychotic discontinuation failed to show a statistically significant effect on global functioning (SMD −0.32; 95% CIs: −0.91, 0.27; I^2^ = 82%) (Table 2; Supplementary Figure 2). To examine the impact of choosing a particular scale when more than one was used, a post hoc meta-analyses was conducted, considering all possible combinations of these measures, which resulted in similar outcomes. The heterogeneity was not explained by any predefined subgroup analysis (Supplementary Table 2). Risk of bias was high for all studies, mostly because of attrition and the lack of a blinded design. Certainty of evidence was very low (Table 2).

### Quality of life

Four RCTs (n = 299 participants) reporting on QoL were identified (Table 2) [6, 9, 35, 41]. RCTs used the Lancashire Quality of Life Profile (LQLP) [41], Subjective Well-being under Neuroleptic Treatment scale [41], European Quality of Life – 5 Dimensions [9], WHO-5 Wellbeing Index [9], WHOQOL [6], and 36-Item Short Form Health Survey questionnaire mental component summary or physical component summary (SF-36 MCS or SF-36 PCS) [35]. Three studies used more than one QoL measure; for the main analysis, we included those that focused exclusively on psychological wellbeing (i.e., LQLP, WHO-5 Wellbeing Index, WHOQOL, and SF-36 MCS) as opposed to those including a component of physical QoL. Using these scales, no significant effect of antipsychotic discontinuation on QoL was detected (SMD −0.11; 95% CIs: −0.34, 0.12; I^2^ = 0%) (Table 2; Supplementary Figure 3). Post hoc analysis using other possible QoL measures and subgroup analysis yielded similar results (Supplementary Table 3). Risk of bias was high for all studies mostly because of the high attrition rate and use of an unblinded design. Certainty of evidence was very low (Table 2).

### Hospital admission

**Figure 3.**
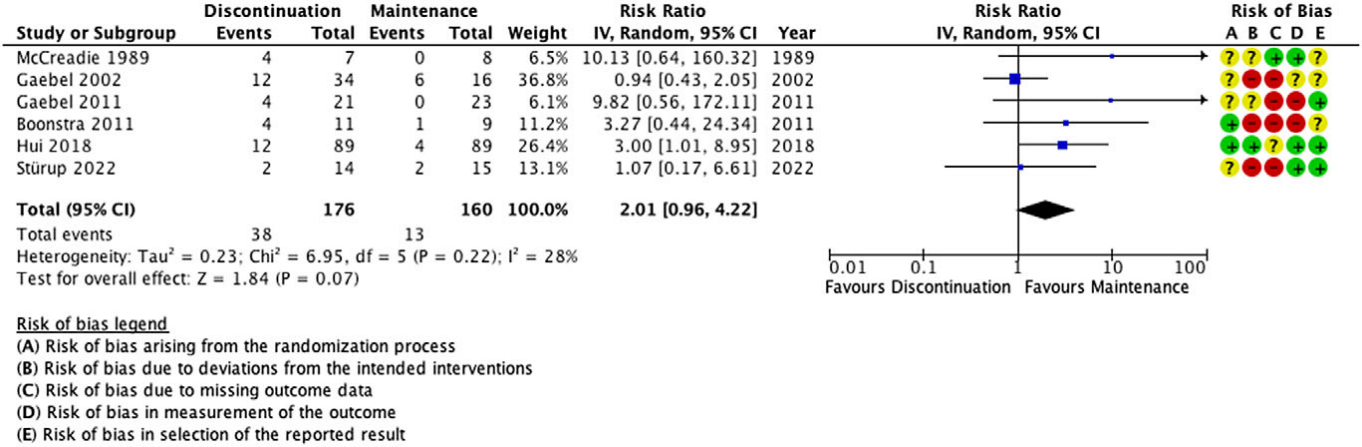
Effect of antipsychotic discontinuation in remitted first-episode psychosis patients on hospital admission. Abbreviations: CI, confidence intervals; IV, inverse variance.

Six RCTs (n = 336 participants) reported hospital admission as an outcome (Figure 3). In one study, the per protocol analysis on hospital admission was extracted instead of the intention to treat because the latter was not available [40]. There was a numerical yet not statistically significant higher risk of rehospitalization in the antipsychotic discontinuation group (RR 2.01; 95% CIs: 0.96, 4.22; I^2^ = 28%). The certainty of evidence was low (Table 2). Risk of bias was high in all studies except two, mainly because of the high attrition rates and because of their unblinded design (Figure 3). Subgroup analyses focusing on the two studies with ‘some concern’ risk of bias (n = 193 participants) showed a significantly increased risk of hospital admission in the discontinuation group (RR 3.54; 95% CIs: 1.28, 9.77; I^2^ = 0%) (Supplementary Table 1). However, this must be interpreted with caution due to the low number of studies in each subgroup of this meta-analysis. The funnel plot shows no sign of publication bias for this outcome (Supplementary Figure 1).

### Symptom severity

Four RCTs (n = 350 participants) reporting on symptom severity assessed with either the PANSS or the SAPS/SANS were identified (Table 2) [6, 9, 35, 41]. Antipsychotic discontinuation did not significantly increase positive symptoms (SMD 0.28; 95% CIs: −0.21, 0.77; I^2^ = 76%), or negative symptoms (SMD -0.03; 95% CIs: −0.36, 0.30; I^2^ = 50%) (Supplementary Figures 4 and 5). Risk of bias was high in all studies except one [35], mainly because of the use of an unblinded design and high attrition rates, as well as of baseline imbalances in mean ratings of negative symptoms for one of them [41]. Subgroup analyses for those two outcomes and did not explain the observed heterogeneity or yield significant results (Supplementary Tables 4 and 5). Overall, the certainty of evidence was very low (Table 2).

### Employment

Two RCTs (n = 242 participants) reported employment as an outcome [6, 35]. No statistically significant effect of antipsychotic discontinuation on employment was observed (RR 1.18; 95% CIs: 0.82, 1.68; I^2^ = 55%) (Table 2). Subgroup analysis was not done because of the low number of studies. Risk of bias was high because of the high attrition rate. The certainty of evidence was very low (Table 2).

### Adverse drug reactions

Five RCTs (n = 392 participants) reporting on ADR were identified [9, 35, 36, 38, 41]. Data from one study could not be used as the results from the discontinuation and maintenance groups were pooled rather than compared [40]. RCTs used the global score or the subscale scores on the Udvalg for Kliniske Undersøgelser (UKU) scale [9, 41], a dichotomized version of the UKU [34], the Liverpool University Neuroleptic Side Effect Rating Scale (LUNSERS) [36], or no validated scale [38]. Overall, the risk of bias was high in four studies because of unsystematic assessment of ADR [38], lack of assessment of ADR if patients discontinued their antipsychotics [9], unblinded design and high attrition rates [41], and unblinded design (Supplementary Figure 7) [36]. The remaining study had low risk of bias [34]. The predefined meta-analysis of this outcome was not possible because only one study reported the presence of ADRs as a dichotomous outcome. Furthermore, post hoc meta-analyses on ADR as a continuous outcome was not performed as the reporting of the ADRs were too heterogeneous. 1. Sedation: One RCT (n = 178 participants) with low risk of bias reported on the occurrence of sedation as an outcome and found significantly less sedation in the discontinuation group than in the maintenance group (49% vs. 70% p = 0.01) [34]. Another RCT (n = 28 participants) reporting on dropouts due to excessive fatigue did not find differences between groups [38]. 2. Weight loss and gastrointestinal ADR: One RCT (n = 178 participants) with low risk of bias reported weight loss, constipation, and reduced salivation as an outcome [34]. More participants in the discontinuation group scored for weight loss (defined as a score of 1 or higher on the UKU) in comparison to the maintenance arm (26% vs. 10% p = 0.01). However, no difference between groups was observed in mean weight and mean BMI at the end of the study. Fewer participants scored for reduced salivation and constipation in the discontinuation group (4% vs. 15% p = 0.04 for both outcomes). 3. Extrapyramidal symptoms: One RCT (n = 28 participants) reported two dropouts in the maintenance group because of tardive dyskinesia and akathisia, respectively [38], and four RCTs (n = 364 participants) used the UKU [9, 34, 41], Extrapyramidal Side Effects scale (EPS) [41], Abnormal Involuntary Movement Scale [34, 41], Barnes Akathisia Rating Scale [34], Hillside Akathisia Scale [41], LUNSERS [36], and/or the Simpson Angus Scale [34] to measure extrapyramidal symptoms. No significant difference was observed between groups in the identified studies. 4. Sexual dysfunction: One RCT (n = 21 participants) reported sexual dysfunction as an outcome with the Change in Sexual Functioning Questionnaire (CSFQ-14 cut off) [9]. Five participants (55.6%) had sexual dysfunction in the discontinuation group and two participants (16.7%) in the maintenance group; no statistical tests were conducted in this study.

## Discussion

The study found that eight RCTs compared the impact of discontinuing versus maintaining antipsychotics after remitted FEP; for our outcomes of interests, the number of available studies varied from 0 for personal recovery to six for hospital admission. All studies were of high risk of bias, except two with some concerns or low risk of bias (depending on the outcome). All outcomes were graded with very low certainty evidence, except for hospital admission that was graded with low certainty evidence. No published RCT reported personal recovery as an outcome. No publication bias was detected in this meta-analysis.

A positive effect of antipsychotic discontinuation on the rates of functional recovery was observed in this meta-analysis. However, it must be interpreted with caution as this evidence is of very low certainty and stems from studies with high methodological biases. First, one of the studies had a dropout rate of almost 20% and assessed functional recovery following a 5.5-year uncontrolled follow-up phase which raises doubts about whether the observed effects can be confidently attributed to the antipsychotic discontinuation. Second, the other clinical trial was terminated prematurely owing to recruitment issues (25 participants as opposed to the intended 250), impacting this study’s statistical power severely. Also, deviations from the intended protocol were observed in the maintenance group of this study. Specifically, 5 out of 15 participants prematurely ceased their medication, and 6 out of 15 gradually tapered their antipsychotics. Such deviations introduce uncertainty into the outcomes of the trial and complicate the interpretation of the results. If antipsychotic discontinuation truly improves functional recovery, defined as a composite outcome of good functioning and symptom remission, an improvement in functioning and/or symptoms would probably also be observed. However, no such improvements were observed in this meta-analysis.

As for the hospital admission outcome, no difference in rates of hospital admission between groups was observed. However, a significant difference was observed in the risk of bias subgroup, with studies using better methodology having a higher risk of hospital readmission following antipsychotic discontinuation. This result is similar to another meta-analysis that reported a small but significant risk difference of 12% [5]. The publication of new evidence explains this slight difference in the results [9].

Similarly, to previously published meta-analysis, no differences between groups were observed for the QoL, employment, global functioning and positive, and negative symptoms outcomes.

As for the effect of antipsychotic discontinuation on ADR, a predefined meta-analysis was not possible due to the heterogeneous reporting of outcomes and only one study reporting ADR as a dichotomous outcome. Indeed, a significant number of RCTs presented ADR using multiple different composite scales as continuous outcomes. No post hoc analysis was performed, as analyzing such different scales would lack clinical relevance and interpretability. Antipsychotic discontinuation reduced sedation, constipation, xerostomia, and increased weight lost in one RCT. Interestingly, this evidence comes from a RCT who used solely quetiapine, which is known, among second-generation antipsychotics, for these types of ADR [43]. No diminution of extrapyramidal symptoms was observed following antipsychotic discontinuation, but this should be interpreted with caution as the risk of bias was high in most studies, some studies did not assess extrapyramidal symptoms adequately, most trials were open label, and one trial used solely quetiapine, a drug known to cause few extrapyramidal symptoms [34].

The weaknesses inherent in this review merit discussion. The heterogeneity in the scales measuring ADRs, QoL, and global functioning limited our ability to pool data effectively. Also, while conducting our search, we identified a failed antipsychotic discontinuation trial (for reasons of security) [31]. However, because it was never published, it was not incorporated into our systematic review. Including unpublished studies in our meta-analysis could alter the observed effects of antipsychotic discontinuation. However, these studies often contain incomplete data and are of lower quality due to the lack of peer review, which could compound the issues of quality, given that most included studies already have high risk of bias.

Many gaps of knowledge remain concerning the decision to discontinue or not antipsychotics following remitted FEP. The effect of this intervention on recovery outcomes is uncertain, and its effects on many other important outcomes are of very low certainty. Furthermore, the effect of antipsychotic discontinuation on most ADRs is unknown. Ongoing trials may solve these gaps in knowledge and aid stakeholders in the shared decision-making process [44, 45]. In the meantime, the use of observational data, although with their inherent biases, could help fill the current gaps of knowledge. Although presenting challenges, the pursuit of further well-designed RCTs to evaluate recovery and other highly regarded patient-reported outcome measures is crucial to enhance the reliability of the present evidence.

## Supporting information

Béchard et al. supplementary material Béchard et al. supplementary material

## Supplementary material

The supplementary material for this article can be found at http://doi.org/10.1192/j.eurpsy.2024.5.
